# Is there a trade-off between economy and task goal variability in transfemoral amputee gait?

**DOI:** 10.1186/s12984-022-01004-8

**Published:** 2022-03-18

**Authors:** I-Chieh Lee, Bretta L. Fylstra, Ming Liu, Tommaso Lenzi, He Huang

**Affiliations:** 1 Joint Department of Biomedical Engineering, North Carolina State University and University of North Carolina at Chapel Hill, Raleigh, NC 27606 USA; 2 Department of Mechanical Engineering and Robotics Center, The University of Utah, Salt Lake City, UT USA

**Keywords:** Mechanical energy, Goal equivalent manifold, Transfemoral amputee, Energy recovery rate, Walking

## Abstract

**Background:**

Energy cost minimization has been widely accepted to regulate gait. Optimization principles have been frequently used to explain how individuals adapt their gait pattern. However, there have been rare attempts to account for the role of variability in this optimization process. Motor redundancy can enable individuals to perform tasks reliably while achieving energy optimization. However, we do not know how the non-goal-equivalent and goal-equivalent variability is regulated. In this study, we investigated how unilateral transfemoral amputees regulate step and stride variability based on the task to achieve energy economy.

**Methods:**

Nine individuals with unilateral transfemoral amputation walked on a treadmill at speeds of 0.6, 0.8, 1.0, 1.2 and 1.4 m/s using their prescribed passive prostheses. We calculated the step-to-step and stride-to-stride variability and applied goal equivalent manifold (GEM) based control to decompose goal-equivalent and non-goal-equivalent manifold. To quantify the energy economy, the energy recovery rate (R) was calculated based on potential energy and kinetic energy. Comparisons were made between GEM variabilities and commonly used standard deviation measurements. A linear regression model was used to investigate the trade-off between R and GEM variabilities.

**Results:**

Our analysis shows greater variability along the goal-equivalent manifold compared to the non-goal-equivalent manifold (p < 0.001). Moreover, our analysis shows lower energy recovery rate for amputee gait compared to nonamputee gait (at least 20% less at faster walking speed). We found a negative relationship between energy recovery rate and non-goal-equivalent variability. Compared to the standard deviation measurements, the variability decomposed using GEM reflected the preferred walking speed and the limitation of the passive prosthetic device.

**Conclusion:**

Individuals with amputation cleverly leverage task redundancy, regulating step and stride variability to the GEM. This result suggests that task redundancy enables unilateral amputees to benefit from motor variability in terms of energy economy. The differences observed between prosthetic step and intact step support the development of prosthetic limbs capable of enhancing positive work during the double support phase and of powered prosthesis controllers that allow for variability along the task space while minimizing variability that interferes with the task goal. This study provides a different perspective on amputee gait analysis and challenges the field to think differently about the role of variability.

**Supplementary Information:**

The online version contains supplementary material available at 10.1186/s12984-022-01004-8.

## Introduction

Energy cost minimization has been widely accepted to regulate gait. Experiments [1, 2] and computational models [3, 4] suggest that individuals select their preferred step length [4, 5], step width [6], step frequency [3], and walking speed [1, 2] to minimize the energy cost of walking. Energy optimization characterizes both normal and pathological gait, acting as an important factor in clinical applications [7, 8]. Individuals with gait impairments operate close to their maximum level of effort and are prone to fatigue even at low walking speeds [9, 10]. Hence, reducing the energy cost of walking is an important goal for rehabilitation [8, 11, 12] and a key metric in assessing the effectiveness of assistive devices such as prostheses, orthoses, and exoskeletons [13, 14].

Optimization principles have been frequently used to describe how individuals adapt their gait pattern. However, there have been rare attempts to assess variability in this gait optimization process. Variability is ubiquitous in motor performance [15, 16]. Yet, a deviation from the average gait pattern might cause a divergence from energy optimality [17, 18]. The fact that variation is inevitable raises a question: Is human variability regulated to assist the energy optimization process? If yes, then how?

Traditionally, movement variability has been linked to noise or error, and a large variability is considered “bad motor control”. This concept might be influenced by traditional statistical and assessment methods of movement variability (e.g., standard deviation) that assume randomness and independence of observations [16, 19]. Opposite to this traditional perspective, variability can be seen to give rise to equifinality, allowing infinite number of ways to perform the same task [20, 21]. Thus, individuals can tolerate movement variability that does not interfere with the task goal without requiring extra effort [22, 23]. These observations suggest that variability and movement redundancy can enable individuals to perform complex tasks reliably and repeatedly, increasing adaptability in motor performance [20, 24].

Researchers have analyzed movement redundancy and variability with different approaches, including uncontrolled manifold (UCM) analysis [24, 25], minimum intervention principle (MIP) [23, 26], and goal equivalent manifold (GEM) [21, 27]. Despite methodological differences, all these approaches decompose the total variability at the body-level into goal-equivalent variability and non-goal-equivalent variability. The goal-equivalent variability is the variability component tangent to the manifold, which does not affect the task goal. The non-goal-equivalent variability is the variability component perpendicular to the manifold, which causes deviation from the task goal. Experiments focusing on quiet standing [24, 28], walking [29, 30], reaching [21, 23], and aiming [27] show that individuals preferentially constrain the non-goal-equivalent variability rather than the goal-equivalent variability, most likely because the latter is not detrimental to the achievement of the task goal. If we assume that non-goal-equivalent variability interferes with the task goal and that this interference requires extra effort to correct, then higher non-goal-equivalent variability should be linked to increased energy cost. Based on this rationale, there may be a trade-off between energy optimization and regulation of task variability.

Understanding energy optimization requires us to determine how the features between the non-goal-equivalent and goal-equivalent variability emerge to reconcile with minimal energy cost. This problem is especially interesting in individuals with unilateral lower-limb amputations, who only have indirect control of the motion of the prostheses. Lower-limb amputees spend more metabolic energy than nonamputee individuals during walking and more proximal amputations are associated with greater metabolic energy cost than distal amputations [12, 31]. The increased energy cost of walking might be related to a lack of ankle push-off power from the prosthesis. Individuals with amputations compensate for the lack of push off power by pulling the thigh forward at the end of stance. This compensatory movement allows for continuous forward propulsion but is highly inefficient [14, 31, 32]. In addition, walking with a passive prosthesis requires greater mechanical work for the step-to-step transition from prosthetic to intact limb, increasing the overall energy cost of walking.

Individuals with unilateral amputation need to adapt at every step to respond to external (i.e., lack of push-off) and internal (i.e., compensatory hip movements) perturbations. Thus, knowing how individuals control the step/stride variability would be beneficial to understand how they achieve optimal energy cost. The purpose of this study is to investigate how individuals with unilateral transfemoral amputation walking with a passive prosthesis regulate step and stride variability. Specifically, we aim to understand how goal-equivalent and non-goal-equivalent variability are regulated with respect to energy economy.

We hypothesize that if individuals with amputations regulate their step/stride variability explicitly to minimize energy cost, then goal-equivalent variability will be larger than non-goal-equivalent variability given that these movement variations do not interfere with task performance. Moreover, we hypothesize that if individuals with amputations regulate their step/stride variability explicitly to minimize energy cost and the variability that interferes with task performance requires extra effort to correct, there will be a trade-off between non-goal-equivalent variability and the energy recovery rate across different walking speeds.

## Methods

### Experimental protocol and data collection

The data collection protocol has been carefully documented in Hood et al. [33], and data has been shared on Springer Nature, scientific data. The present study examined unilateral amputees walking on the treadmill who comfortably walked at or above 0.8 m/s and were classified as full community ambulators (K3) [33]. Seven male and two female transfemoral amputees (39.56 ± 12.5 years old) were recruited in this study. Participant inclusion criteria included the following: age between 18 and 85 years, at least 6 months after amputation, daily use of their prescribed prosthesis, and ability to walk on a treadmill. Exclusion criteria included serious comorbidities (including musculoskeletal, cardiac, neuromuscular, skin, or vascular conditions) and inability to communicate and/or be understood by investigators. The institutional review board at the University of Utah (IRB00099066) and the US Human Research Protection Office (HRPO) of the US Army Medical Research and Development Command (HSRRB log number: A-19840) approved the study protocol. Participants provided written informed consent to participate in the study. Participants also provided written consent for the use of pictures and videos of the experiments. The details of the demographic data are provided in Table 1. Upon enrollment, subjects were asked about their previous experience with walking on a treadmill. Subjects who reported using the treadmill regularly were asked to report their maximum comfortable treadmill speed and reliance on handrails during treadmill use. If a subject reported little to no experience walking on a treadmill with a prosthesis, they were provided training. Training consisted of the subject walking on the treadmill for 2–5 min intervals with periods of rest in between each interval. During each training session, the experimenter started at the slowest speed of the treadmill (i.e., 0.2 m/s) and slowly increased speed until the subject reported that the speed was their maximum comfortable treadmill speed. Each training session lasted less than 2 h. Weekly training sessions were conducted until no further improvements in comfortable walking speed were observed. Before the start of the data acquisition session, subjects were given time to familiarize with all tested speeds.

**Table 1:** Subject information

Subject	Age	Gender	Mass (kg)	Height (m)	Amputation side	Cause of amputation	Age of amputation	Knee prosthesis	Foot prosthesis	Socket suspension
TF01	26	Male	64.9	1.78	Right	Traumatic	5	Plie FI	AllPro FI	Suction
TF02	49	Male	102.1	1.91	Left	Traumatic	10	C-Leg Obk	Triton Obk	Pin lock
TF03	42	Male	95.3	1.85	Right	Traumatic	6	Rheo Os	AllPro FI	Suction
TF04	51	Male	70.3	1.68	Right	Traumatic	33	C-Leg Obk	Trias Obk	Suction
TF05	61	Male	88.5	1.88	Left	Traumatic	3	Rheo Os	Proflex XC Os	Vacuum
TF06	23	Female	68.0	1.75	Right	Traumatic	5	Plie FI	Proflex XC Os	Suction
TF07	36	Male	100.2	1.80	Left	Traumatic	8	3R80 Obk	AllPro FI	Suction
TF08	38	Male	104.3	1.91	Left	Traumatic	33	Plie FI	Soleus ClgPk	Suction
TF09	30	Female	59.0	1.60	Left	Traumatic	10	3R80 Obk	AllPro FI	Lanyard

*Obk* Ottobock, *FI* freedom innovations LLC, *Os* Össur

Each participant was asked to walk on a treadmill with their daily passive prostheses at speeds of 0.6, 0.8, 1.0, 1.2 and 1.4 m/s. During acceleration and deceleration of the treadmill, participants were instructed to hold on to the treadmill handrails. After the treadmill reached the constant speed, the participants were encouraged to walk without holding the handrails and then recording started. For each treadmill speed, five trials contained around 10 continuous strides except for TF01 having four trials at 0.6, 0.8 and 1.0 m/s and TF02 having 4 trials at speed 0.6 m/s. Only TF05 and TF06 held the handrails all the times during the 1.4 m/s condition. During walking, a 10-camera motion capture system (Vicon, Oxford, UK) was used to record the reflective markers position at 200 Hz and a split-belt Bertec fully instrumented treadmill (Bertec Co; Columbus, OH, USA) was used to record the bilateral ground reaction forces at 1000 Hz. Reflective markers were placed on the participants following a modified Plug-in-Gait Model [34, 35]. Marker trajectories and ground reaction force data were synchronized, recorded, and pre-processed using Vicon Nexus 2 software. A low-pass Butterworth filter with a cut-off frequency of 6 Hz was applied for the marker trajectories. Inverse dynamic of rotational, translational, and potential energy of 15 segments (head, thorax, pelvis, and left and right forearms, upper arms, hands, thighs, shanks, feet) were calculated using Visual 3D. Anthropometric measurements included body weight, height, and segment lengths were taken from each participant to accurately reconstruct the representative model. For each segment, the COM location, the radius of head, and the base radii of the other segments were estimated based on the anthropometric dimension of the 50 percentile composite subjects from Hanavan et al. [36]. The segment mass for each participant was calculated as a percentage of whole-body mass based on de Leva [37], and the mass of the prosthetic ankle and foot was the actual measured mass.

### Data analysis

**Fig. 1:**
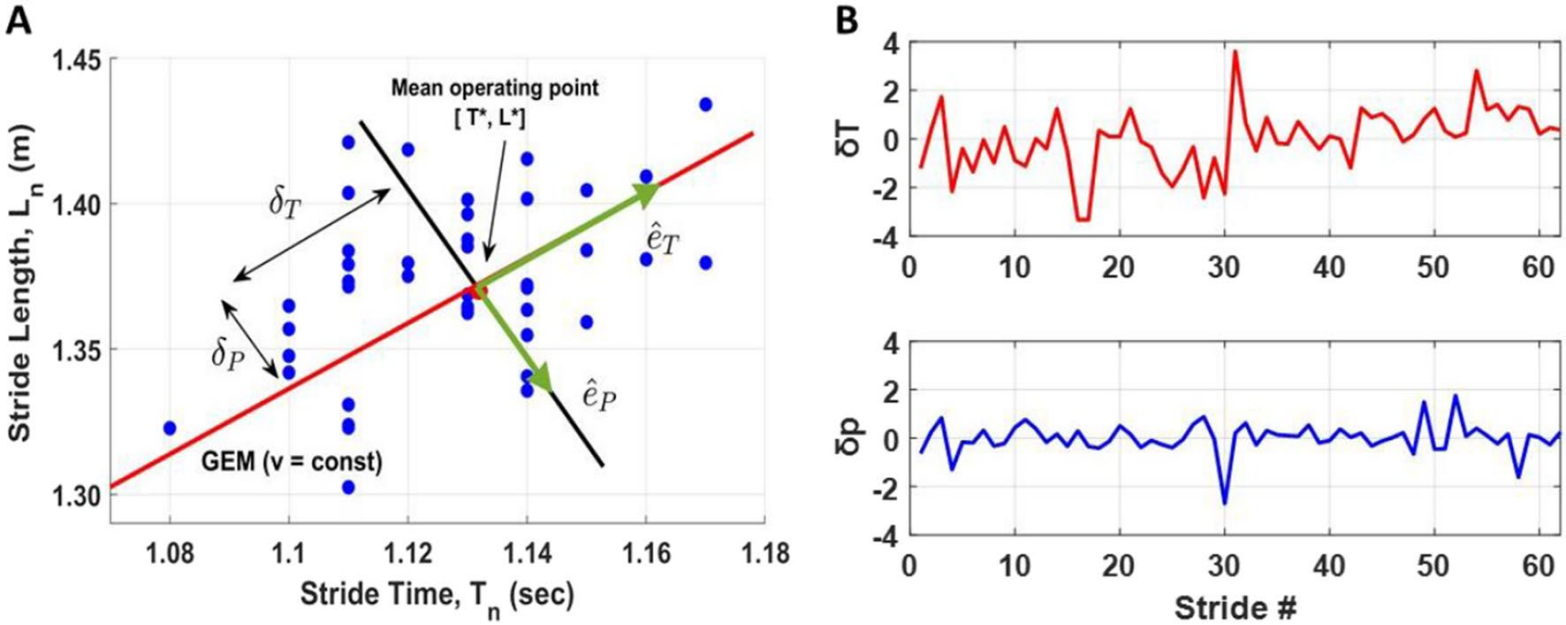
**A** Schematic depicting the goal equivalent manifold (GEM) for maintaining constant walking speed (v) using data from TF01. The red dot is the preferred mean operating point. Each blue dot represents the combination of stride time and stride length for an amputee participant with walking speed at 1.2 m/s. Dots that lie exactly on the red diagonal line (GEM) achieved the same speed and satisfied the goal. **B** The time series of δ_T_ and δ_p_ deviations for the data set shown in **A**

**Fig. 2:**
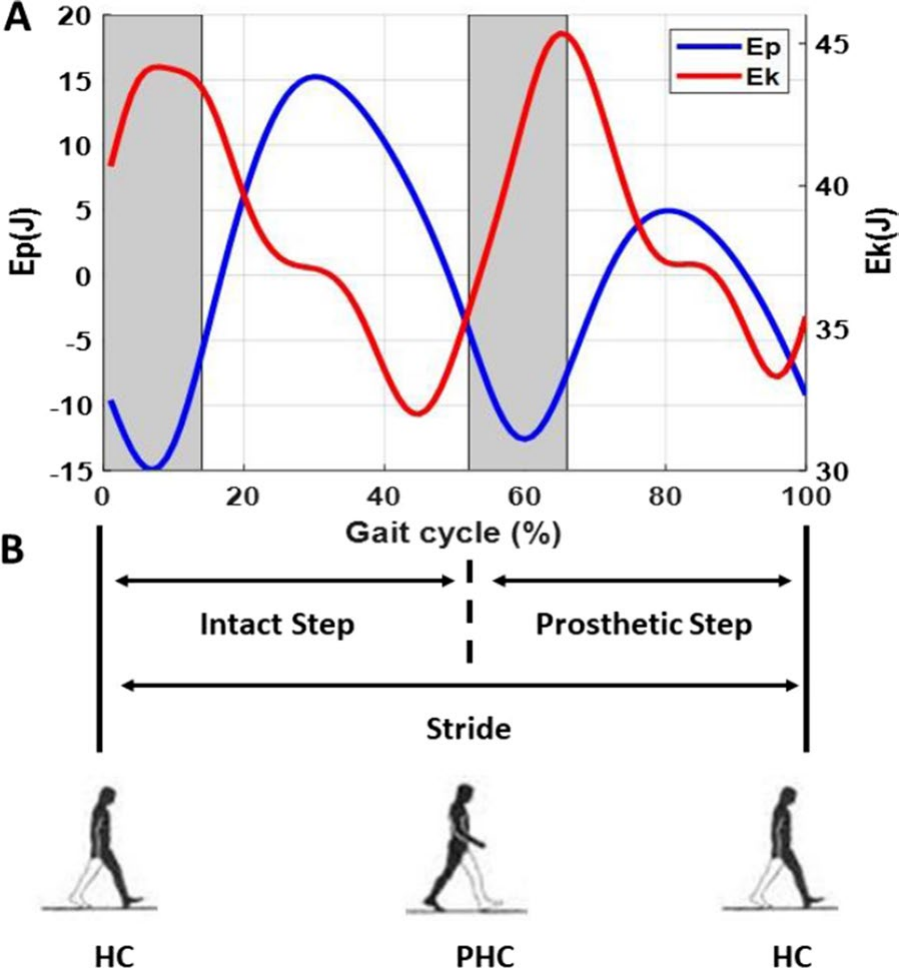
**A** Example of ${\mathrm{E}}_{p}$ and ${\mathrm{E}}_{k}$ oscillations are plotted as a function of gait cycle duration. The shaded area indicates the periods of double support. The plot shows an out-of-phase oscillation of kinetic and potential energy, allowing energy exchange to occur between ${\mathrm{E}}_{k}$ and ${\mathrm{E}}_{p}$. **B** Demonstration the stride and step (prothesis/intact step) period determined in this study. HC is heel contact of intact limb; PHC is heel contact of prosthetic limb. Stride period is determined from HC to the next HC. Prosthetic step is determined from PHC to HC where prosthetic limb leads, intact limb trails, and single support is on the prosthetic limb. Intact step is determined from HC to the PHC where intact limb leads, prosthetic limb trails, and single support is on the intact limb

We applied GEM-based control to decompose the goal-equivalent and non-goal-equivalent variability [30, 38] (details see Fig. 1 and “Methods”). To quantify the energy economy, we calculated the energy recovery rate for each step based on potential energy (${E}_{p}$) and kinetic energy (${E}_{k}$) [39]. To optimize the recovery of mechanical energy, the ${E}_{p}$ and ${E}_{k}$ curves must have the same shape, be equal in amplitude, and be opposite in phase, as in a pendulum. A higher energy recovery rate indicates more efficient walking (see Fig. 2B and “Methods” for details).

Stride-to-stride and step-to-step analysis were both performed in this study. For each stride, n, stride time (T_n_) was taken as the time between consecutive right heel strikes. Stride length (L_n_) was taken as the anterior–posterior distance travelled during each stride using the position of right and left heel markers. Stride speed (S_n_) was calculated as S_n_ = L_n_/T_n_. Step analysis was calculated using the same methods and taken the time and length between heel contact to the contralateral side heel contact. Prosthetic step was determined from prosthetic heel contact to intact heel contact where the intact limb provided push-off power during the initial double support phase and then transferred to the prosthetic single support phase. Intact step was determined from intact heel contact to the prosthetic heel contact where the prosthesis leg provided push-off power during the initial double support phase and then transferred to the intact leg single support phase. (see Fig. 2B).

### Goal equivalent manifold (GEM)

We applied a GEM based method to decompose the goal-equivalent and non-goal-equivalent variabilities on treadmill walking as originally proposed by Dingwell [38]. The primary goal for treadmill walking with speed *v* is to not walk off the treadmill. Thus, we can define the task goal specifically considered in the sagittal plane. The treadmill walking task is specified by an *inequality constraint* of the form

1 $$\left|\sum_{n=\mathrm{0,1},2\dots m}^{m}\left({L}_{n}-v{T}_{n}\right)\right| \le K is true for any m < N,$$

where *L*_*n*_ and *T*_*n*_ are the stride length and time, respectively, at stride *n*; *v* is the treadmill speed; *K* is nominally half the treadmill’s length; m is any number of stride smaller than N; and *N* is the total number of strides. The simplest strategy to satisfy Eq. (1) is to keep *v* constant at each step, which was formulated using the goal function:

2 $${L}_{n}-v{T}_{n}=0 \to \frac{{L}_{n}}{{T}_{n}}=v.$$

This equation is equivalent to a strategy of matching the treadmill speed at *each* stride. Due to the body redundancy, there are many possible gait strategies that one *could* use to satisfy this requirement, including a variety of “drunken” or “silly” walks.

Hence, all [${L}_{n}$, ${T}_{n}$] pairs that satisfied Eq. (2) defines the GEM, which was a red solid line in the ${L}_{n}$ versus ${T}_{n}$ plane (see Fig. 1A).

To analyze walking dynamics relative to the GEM, we first normalized each ${L}_{n}$ and ${T}_{n}$ to unit variance ($\sigma =1$) to provide an intuitive reference for comparison by dividing its own standard deviation:

3 $$\stackrel{\sim }{{T}_{n}}={T}_{n} /\sigma \left({T}_{n}\right) and \stackrel{\sim }{{L}_{n}}={L}_{n} /\sigma ({L}_{n}).$$

This yielded a GEM defined by the dimensionless walking speed: $\tilde{v }=mean(\stackrel{\sim }{{L}_{n}} /\stackrel{\sim }{{T}_{n}})$. We then define the new coordinates centered at a mean “preferred operating point”: [$\stackrel{\sim }{{T}_{n}}$*, $\stackrel{\sim }{{L}_{n}}$*] = [mean($\stackrel{\sim }{{T}_{n}}$), $\tilde{v }\stackrel{\sim }{{T}_{n}}$*] and re-expressed as ${\stackrel{\sim }{{T}_{n}}}^{^{\prime}}= \stackrel{\sim }{{T}_{n}}$ − $\stackrel{\sim }{{T}_{n}}$* and ${\stackrel{\sim }{{L}_{n}}}^{^{\prime}}= \stackrel{\sim }{{L}_{n}}$ − $\stackrel{\sim }{{L}_{n}}$*.

Finally, the goal-equivalent deviation along the GEM (δ_T_ is the goal-equivalent deviation), and non-goal-equivalent deviations perpendicular to the GEM (δ_p_ is the non-goal-equivalent deviation) were calculated as (see Additional file 1: Appendix for the derive process):

4 $$\left[\begin{array}{c}{\delta }_{T}\\ {\delta }_{p}\end{array}\right]= \frac{1}{\sqrt{1+{v}^{2}}} \left[\begin{array}{cc}1& v\\ -v& 1\end{array}\right] \left[\begin{array}{c}{\stackrel{\sim }{{T}_{n}}}^{^{\prime}}\\ {\stackrel{\sim }{{L}_{n}}}^{^{\prime}}\end{array}\right].$$

To quantify how variability was distributed relative to the GEM, the standard deviation of each new time series (δ_T_ and δ_p_) was calculated (see Fig. 1B).

Assuming that the participant intent is to walk in a funky gait pattern, this intention is nested within the defined goal function and the modification of stride time and stride length due to the deviation of the position or the strategy will be reflected on the quantified goal-relevant and goal-irrelevant variabilities.

### Energy recovery rate

The potential and kinetic energy of the whole body, divided unto *s* segments of mass *m*, can be measured from the gravitational and the kinetic energy of each segment calculated at each instant of time (*t*) relative to the frame of reference (Fig. 2):

The whole-body potential energy was calculated as:

5 $${\mathrm{E}}_{p}(\mathrm{t})= \sum_{i=1}^{s}\left({m}_{i}g{h}_{i}\left(t\right)\right),$$

where h_i_ and m_i_, respectively, are the vertical distance of center of mass to the ground and the mass of the ith segment, relative to the frame of global reference; $g$ is the gravitational acceleration (9.81 m/s).

The whole-body kinetic energy was calculated as:

6 $${E}_{k}\left(t\right)= \sum_{i=1}^{n}(\frac{1}{2}{m}_{i} {[v}_{xi}^{2}\left(t\right)+{v}_{yi}^{2}\left(t\right)+{v}_{zi}^{2}\left(t\right)]+ \frac{1}{2}{m}_{i} {[k}_{xi}^{2}{\omega }_{xi}^{2}(t)+{k}_{yi}^{2}{\omega }_{yi}^{2}\left(t\right)+{k}_{zi}^{2}{\omega }_{zi}^{2}(t)]),$$

where ${v}_{i}$, is the linear velocity relative to the frame of the global references in x, y and z axis; ${k}_{i}$ and ${\omega }_{i}$, respectively, are the radius of gyration about the axis through the center of mass and angular velocity of the ith segment (frame of local reference). 

Thus, the total energy can be computed as:

7 $${\mathrm{E}}_{\mathrm{total}}\left(\mathrm{t}\right)= {\mathrm{E}}_{p}\left(\mathrm{t}\right)+{\mathrm{E}}_{k}\left(\mathrm{t}\right).$$

Over the stride and step period, the energy exchange over the stride and step period was computed according to Winter [39]. The external work (${w}_{ext}$) on the COM during the N sample period was computed as:

8 $${w}_{ext}=\sum_{i=1}^{N}\left(\left|\Delta {E}_{p}+\Delta {E}_{k}\right|\right).$$

Assuming no energy exchanges between ${E}_{k}$ and ${E}_{p}$, the work (${w}_{net}$) done by a segment during N sample periods is:

9 $${w}_{net}= \sum_{i=1}^{N}\left(\left|\Delta {E}_{p}\right|+\left|\Delta {E}_{k}\right|\right),$$

where the $\Delta {E}_{p}$ and $\Delta {E}_{k}$ is the energy difference between two successive time points.

The energy recovery rate (R) represents the percentage of mechanical energy recovery via exchange between kinetic and potential energy in the COM movement. This is computed as:

10 $$R=100 \cdot \frac{({{w}_{net}-w}_{ext}) }{{w}_{net}}.$$

In an ideal energy recovery mechanism, the work associated with changes of potential energy is exactly the same as the work associated with kinetic energy changes, but with different sign: W_p_ = − W_k_. That means that work produced to increase the potential energy can be obtained by reducing the kinetic energy and can again be returned to increase the kinetic energy at the next step-to-step transition. Hence, the larger value of R indicates better energy economy.

### Statistics

We first performed one-way repeated ANOVA to examine the commonly used gait features across walking speeds (mean and standard deviation (SD) of stride/step length and time). The trend of the effect on speed was tested using within-subject contrast on a linear, quadratic, cubic, and order 4. To answer the question of the first hypothesis, the differences between the two types of decomposed variabilities (δT and δp) across five walking speeds were tested using 2-way repeated ANOVA (2 variabilities × 5 speeds) in stride, intact step, and prosthesis step. To answer the second question, we performed a simple linear regression of energy recovery rate (R) as the dependent variable and δp and walking speed as the independent variable for each stride, prosthetic step, and intact step (model: R_i_ = β0 + β1 δp_i_ + β2 speed_i_ + ε_i_). If the coefficient of δp is negative and reaches a significant level, it indicates a trade-off between R and δp. To understand the role of push-off for prosthetic and intact leg in respect to the energy exchange, a follow-up analysis was also conducted. We performed 2-way repeated ANOVA (2 (intact & prosthesis step) × 5 (Speeds)) to compare the energy recovery rate between step types and speeds. The significant level was set at α = 0.05. For the main effects that reached the significance level, the Bonferroni test was used for the post hoc comparisons. The Shapiro–Wilk Test was used to test the normality prior applying the ANOVAs and all the tests did not reach significant difference.

## Results

### Primary gait features across walking speeds

**Fig. 3:**
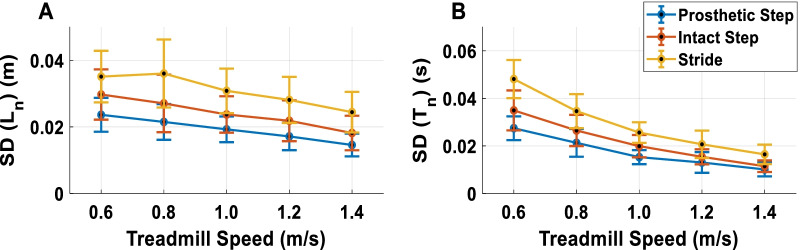
Primary gait parameters. Standard deviations (**A**, **B**) for stride and step length (Ln) and time (Tn) as a function of walking speed from 0.6 m/s to 1.4 m/s. Error bars indicate between subject 95% confidence intervals at each speed. The SD of length and the SD of time in stride and step length linearly decreased along with speed

Figure 3A, B depict the standard deviations (SD) of the length and time of prosthetic step, intact step, and stride, respectively. For the length SD of prosthetic step, intact step, and stride linearly decreased with faster walking speed (Fig. 3C) (prosthetic step: F(4,32) = 11.629, p < 0.001, $\eta_{p}^{2}$ = 0.592, linear effect F(1,8) = 24.876, p < 0.001, $\eta_{p}^{2}$ = 0.757; intact step: F(4,32) = 6.826, p < 0.001, $\eta_{p}^{2}$ = 0.460, linear effect F(1,8) = 22.238, p < 0.001, $\eta_{p}^{2}$ = 0.735; stride: F(4,32) = 6.646, p < 0.001, $\eta_{p}^{2}$ = 0.454, linear effect F(1,8) = 14.889, p < 0.01, $\eta_{p}^{2}$ = 0.650). The time variability of prosthetic step, intact step, and stride also linearly decreased with faster walking speed (Fig. 3D) (prosthetic step: F(4,32) = 51.593, p < 0.001, $\eta_{p}^{2}$ = 0.866, linear effect F(1,8) = 158.259, p < 0.001, $\eta_{p}^{2}$ = 0.9527; intact step: F(4,32) = 29.234, p < 0.001, $\eta_{p}^{2}$ = 0.785, linear effect F(1,8) = 51.545, p < 0.001, $\eta_{p}^{2}$ = 0.866; stride: F(4,32) = 53.982, p < 0.001, $\eta_{p}^{2}$ = 0.871, linear effect F(1,8) = 117.232, p < 0.01, $\eta_{p}^{2}$ = 0.936).

### Gait variability decomposed based on GEM

**Fig. 4:**
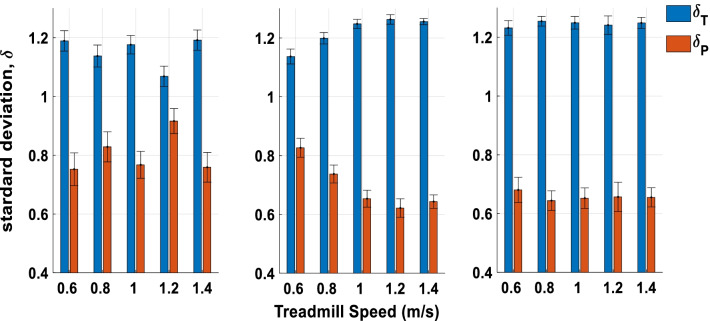
GEM-Based Decomposition of Gait Variability. **A** Standard deviations for all δ_T_ and δ_p_ time series at all 5 walking speeds calculated from each stride period. **B** Standard deviations for all δ_T_ and δ_p_ time series at all 5 walking speeds calculated from each prosthetic step. **C** Standard deviations for all δ_T_ and δ_p_ time series at all 5 walking speeds calculated from each intact step

The first hypothesis was whether amputees exhibited greater variability along the GEM (task goal) rather than perpendicular to it. Figure 4A–C, respectively demonstrate that the task relevant (δ_T_) variability was significantly larger than task irrelevant (δ_P_) variability in stride, prosthetic step, and intact step (stride: F(1,8) = 47.885, p < 0.001, $\eta_{p}^{2}$ = 0.857; prosthetic step: F(1,8) = 158.198, p < 0.001, $\eta_{p}^{2}$ = 0.952; intact step: F(1,8) = 172.667, p < 0.001, $\eta_{p}^{2}$ = 0.956). For the interaction effect between speed and variability, there was a significant difference in prosthetic step (F(4,32) = 6.827, p < 0.001, $\eta_{p}^{2}$ = 0.46) and a marginal level on stride (F(4,32) = 2.670, p = 0.051, $\eta_{p}^{2}$ = 0.26). The post hoc analyses for δ_T_ in prosthetic step showed that at the speed of 1.0 m/s, 1.2 m/s and 1.4 m/s were equal but were significantly higher than the speed at 0.6 and 0.8 m/s. δ_p_ in prosthetic step, on the other hand, showed a trend as a U shape that walking speed at 1.0 m/s was significantly smaller than 0.6 m/s and 0.8 m/s and significantly smaller than 1.2 m/s and 1.4 m/s. The post hoc analyses for δ_T_ in stride showed that 1.2 m/s was significantly smaller than 1.0 m/s and 1.4 m/s, and δ_p_ showed 1.2 m/s was significantly larger than 1.0 m/s and 1.4 m/s.

### Energy recovery rate

**Fig. 5:**
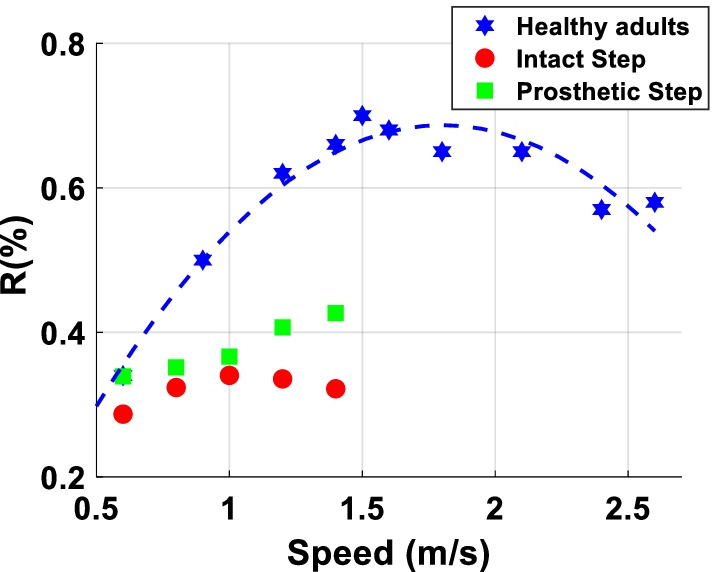
Energy recovery rate (R) are plotted as a function of walking speed for healthy adults (hexagram), Intact Step (circles) and Prosthetic Step (square). Adult data were reproduced from Willems et al., 1995

Figure 5 depicts the energy recovery rate of prosthetic step and intact step. There was a significant interaction between step types and speed (F (4,32) = 5.552, p = 0.002, $\eta_{p}^{2}$ = 0.40), and the post hoc analysis indicated only in prosthetic step at speeds 1.2 m/s and 1.4 m/s were significantly larger than other speeds. The second hypothesis was whether there is a trade-off between δp and energy recovery rate (negative relationship). We performed a linear regression between δp, speed, and energy recovery rate of prosthetic step and intact step. The results showed the coefficient in prosthetic step and intact step reached significant differences with a negative coefficient (see Table 2).

**Table 2:** The results of the linear regressions of for energy recovery rate of prosthetic step and intact step as a function of δp and speed (model: R_i_ = β0 + β1δp_i_ + β2 speed_i_ + ε_i_)

	Coefficient	Standard error	p
Prosthetic step			
β0	0.51	0.07	< 0.001*
Speed	0.06	0.05	0.18
δp	− 1.78	0.16	< 0.001*
Intact step			
β0	0.31	0.07	< 0.001*
Speed	0.14	0.05	0.02*
δp	− 1.20	0.17	< 0.001*

## Discussion

The purpose of this study was to investigate how individuals with unilateral transfemoral amputation regulate step and stride variability on the goal-equivalent and non-goal-equivalent manifold to achieve energy economy. We examined the hypothesis that if amputees are regulating variability in terms of task goal, a large portion of goal-equivalent variability would be observed. In addition, we examined whether there would be a trade-off between non-goal-equivalent variability and energy recovery rate across different walking speeds under the assumption that individuals require more energy to correct for the non-goal-equivalent variability.

Looking at the main results of primary gait features, the standard deviation of stride/step length and time linearly decreased with faster walking speeds (Fig. 3A, B). This trend was consistent for both prosthesis steps and intact steps. While the standard deviation quantified the magnitude of deviation from the mean of all strides/steps, the time series of goal-equivalent (δ_T_) and non-goal-equivalent (δ_p_) variabilities exhibited the step-to-step change in respect to the task goal with the temporal order. δ_T_, qualitatively, showed larger amplitudes than the δ_p_ (Fig. 1B) and the variabilities of δ_T_ were significantly higher than the δ_p_ across all speeds (Fig. 4). These results support our first hypothesis and indicate that amputees explicitly regulate step/stride variability tuned to the goal equivalent manifold (GEM). Our findings on unilateral amputees followed the same trend as healthy young adults [38, 40] and elderly [41, 42]. The ability to leverage the task redundancy could be critical when tasks become more demanding. For example, walking on uneven terrain requires individuals to adjust each step rather than change their average gait pattern. In addition, this concept can be used as a guiding principle for designing fault-tolerant controllers for powered prostheses. To achieve this goal, a powered prosthesis controller should minimize the δ_p_ and only correct errors distributed on the δ_p_ because these errors interfere with task performance. In contrast, errors that vary along with the δ_T_ should be tolerated as they do not interfere with the task performance. A prosthesis controller following this principle may improve safety while reducing control effort.

The goal-equivalent (δ_T_) and non-goal-equivalent (δ_p_) variabilities demonstrated a different speed effect compared to the standard deviation of stride/step length and time, and such effects reflect the unique characteristics of amputee gait (Fig. 4). During the prosthetic step, the intact leg regulates the push-off power, which propels the body forward, and the swing movement. Thus, the intact leg largely determines the step time and step length. Our results show that the variability of δ_T_ and δ_p_ changed with walking speed forming an inverted U shape and U shape, respectively (Fig. 4B). A similar trend was observed with young and elderly healthy adults [38, 40, 43]. This trend suggests that the intact leg can adjust the δ_T_ and δ_p_ variabilities that tuned to the preferred walking speed for unilateral amputees (around 1 m/s) [44]. Interestingly, the intact step did not show the same adaptability to walking speed. During the intact step, the passive prosthetic leg provides limited push-off power [32, 45] and swing time adaptability. Thus, although microprocessor-controlled knee prostheses can adjust the joint damping to walking speed [46, 47], our results indicate that this adaptation mechanism is not enough to achieve similar task performance to the intact leg. Future work could test whether the U-shaped relationship observed in the intact leg occur in powered prostheses capable of imitating the intact-leg push-off power and swing time adaptability.

In this study, we quantified energy recovery rate at each step based on potential energy (${E}_{p}$) and kinetic energy (${E}_{k}$) [39]. Previous studies have assessed the effectiveness of the pendulum mechanism during human walking by measuring the fraction of the total mechanical energy that is recovered as a result of the transduction between ${E}_{p}$ and ${E}_{k}$ [48, 49]. To optimize the recovery of mechanical energy, which leads to the most efficient walking pattern, the ${E}_{p}$ and ${E}_{k}$ curves must have the same shape, be equal in amplitude, and be opposite in phase, as in a pendulum. A higher energy recovery rate indicates more efficient walking (see Fig. 2B and “Methods” for details). Previous studies showed that for healthy adults at preferred walking speed (~ 1.6 m/s), as much as 70% of the required external mechanical energy can be recovered due to this energy saving mechanism [48, 50, 51]. The other 30% of external mechanical energy is lost from the system and must be supplied by the muscles [52]. Our study shows that unilateral amputees have lower recovery rate than healthy adults. At 1.4 m/s, the average energy recovery rate during the prosthetic step was about 40%. Moreover, the recovery rate could be up to 60% depending on the amputee individual (see Fig. 5). This result explains why individuals with amputation using a passive prosthesis spend, on average, more energy during walking than nonamputee individuals.

As expected, the prosthetic step had a larger energy recovery rate than the intact step. Interestingly, the speed trends for the prosthetic and intact steps were different. The prosthetic step showed a linearly increase of the energy recovery rate with walking speed, whereas the intact step showed an inverted U-shaped profile. This difference in the speed trends could be due to the different ability of the intact and prosthetic leg to generate positive work during push-off. Previous studies in healthy adults found an inverted U-shaped relationship, although for a wider range of walking speeds than the one used in this study (i.e., 0.5–2.5 m/s vs. 0.8–1.4 m/s) [48, 50, 51]. In these studies, energy recovery rate gradually increased from 0.6 m/s to 1.6 m/s, which is similar to the walking speed range of our study. This result is in agreement with our finding in the prosthetic side.

Previous studies in nonamputee individuals show that, at low walking speeds, the potential energy changes are larger than the kinetic energy changes (see Fig. 5). When walking speed increases, the kinetic energy changes increase relative to the potential energy changes, exceeding the potential energy changes after the walking speed is above the preferred walking speed. Thus, the energy recovery rate reaches its maximum for the self-selected speed and it is lower than its maximum both above and below the self-selected speed [48]. Our study shows that when the intact leg is in charge of forward propulsion (i.e., prosthesis step), the amplitude of kinetic energy continuously increases along with walking speed. However, when the prosthetic leg is in charge of forward propulsion (i.e., intact-leg step), the capacity of increasing the amplitude of kinetic energy relative to the potential energy reaches a limit after the walking speed is above the amputee’s preferred walking speed. We speculate that this inability to increase the amplitude of kinetic energy might be due to the passive prosthesis generating less power than the intact leg during push-off, resulting in net negative work during the intact step [32, 45, 53]. The increase of energy dissipation during double support phase at fast speed might explain the inverted U-shaped relationship in a smaller range of walking speed. We speculate that, with proper control of propulsive torque, powered prostheses [14, 45] could enable amputees to achieve better energy recovery rate. Future studies should investigate the step-to-step change of energy recovery rate with powered prostheses to test this hypothesis.

The second hypothesis was tested by applying linear regression to the time series of δ_p_ and R calculated extracted from each stride and step. Using this regression, we found that adding the δ_p_ in the linear model significantly explains the energy recovery rate. Moreover, the negative coefficient of the linear regression supports the trade-off relationship between δ_p_ variability and recovery rate. Since the fitting considered the temporal order with δ_p_ and the energy recovery rate, this result demonstrates that individuals with unilateral amputations leverage movement redundancy to correct small deviations at each step to control the task variability while minimizing energy cost. This short-term control mechanism (step-to-step) exploits the inherent task redundancy enabling unilateral amputees to receive potential benefits from motor variability in achieving energy economy. This result could inform a new assessment method for the functionality of powered lower-limb prostheses. For example, better prosthesis designs would be reflected in the GEM analysis by showing a larger δ_T_ than δ_p_ as it indicates a better energy recovery rate. This assessment method could be the object of future work for rehabilitation engineers.

We recognize that the energy recovery rate might not accurately estimate the energy cost of walking, especially for fast walking speeds [32]. Although measuring the oxygen consumption can lead to a more accurate estimate of the energy cost of walking, it cannot provide step-to-step cost, which is needed for the proposed analysis of GEM variabilities. Using more elaborate musculoskeletal models might provide a better estimation of the energy cost of walking; however, inverse dynamics calculations have been shown not to solve this problem easily [54] and the current validity of more elaborate musculoskeletal models does not warrant success with such an approach either. In addition, previous studies have shown that the effect of speed on the oxygen consumption showing an inverted-U shape turned to the amputees’ preferred walking speed [10, 55]. Considering the average ${\delta }_{P}$ in Fig. 4, we postulated that under a longer time-scale, the trade-off between ${\delta }_{P}$ and oxygen consumption will still hold at least for the prosthetic side. We believe the interaction of task variability and energy recovery rate could provide some clarification on the issue. Further interpretation would be highly speculative and require more studies.

In this study, we defined the goal function under the rationale of treadmill walking with a constant speed. However, one of the limitations of this study is that the goal (maintaining walking speed) may not be what the participants intend to do; instead, it is the necessary criteria to perform the task. This characteristic provides us a simple way to capture the goal equivalent manifold as well as to decompose the variabilities. Assuming the participant has the intent to walk in a “funky” gait pattern, this intention is nested within the defined goal function and the modification of stride time and stride length due to the deviation of the position or the strategy will be reflected on the quantified goal-equivalent and non-goal-equivalent variabilities. However, we do not know if the task goal was indeed the goal adopted by the human subjects. It is an interesting question for a future study to investigate how the task goal and human goal intervene to regulate step and stride variabilities on the GEM. Moreover, treadmill walking is a well-defined task where it is easy to define the task goal. Considering the variety of tasks in clinical settings (e.g., walking, running, stair climbing, etc.), it is not clear how to systematically define these task goals to decompose the goal variabilities, and thus limited the application of the GEM.

In addition, this study recruited nine transfemoral amputees. Even though all of them are K3 level, their demographic data are widely spread (e.g., age, gender, proscribed prosthesis, etc.). Also, we did not record participants’ preferred walking speed and that prevents us to investigate this effect associated with energy cost and variabilities. Thus, future study could expand the sample size and consider the demographic analysis into the model fitting to improve the conclusion and take into account the preferred walking speed into consideration.

To the best of our knowledge, this is the first study to analyze the gait variability at the level of task goal in connection to energy optimization in individuals with lower-limb amputations. Given that human behavior is goal driven, instead of focusing on common gait features such as step mean and standard deviation, we took the task goal into consideration. We believe that the results of this study will provide a different perspective on amputee gait analysis and challenge the field to think differently about the role of variability.

## Conclusion

This study investigated how individuals with unilateral transfemoral amputation walking with a passive prosthesis regulate step and stride variability on the goal-equivalent and non-goal-equivalent manifolds. Our results suggest that individuals with amputation cleverly leverage task redundancy, regulating step and stride variability to the goal equivalent manifold (GEM). This result suggests that task redundancy enables unilateral amputees to benefit from motor variability in terms of energy economy. The differences observed between prosthetic step and intact step support the development of prosthetic leg capable of enhancing positive work during the double support phase. Moreover, the results of this study motivate the development of powered prosthesis controllers that allow for variability along the task space while minimizing variability that interferes with the task goal. This study provides a different perspective on amputee gait analysis and challenge the field to think differently about the role of variability.

## Supplementary Information

**Additional file 1: **Derivation of the Equation 4.

## Data Availability

The data has published at scientific data.
