# Supplementary material for: Is there a trade-off between economy and task goal variability in transfemoral amputee gait?
Source: J Neuroeng Rehabil. 2022 Mar 18;19:29. doi: 10.1186/s12984-022-01004-8 (PMC8932056; doi:10.1186/s12984-022-01004-8)
Supplement: Supplementary file 1 — Additional file 1: Derivation of the Equation 4. [file 12984_2022_1004_MOESM1_ESM.docx]

Appendix

Derivation of the Equation 4

It is a coordinate geometry problem to derive the $\left[ \begin{matrix} \delta_{T} \\ \delta_{p} \end{matrix} \right]$. We will reach the equation 4 in two steps. First, we recenter the coordination system to the operating point, P$(\tilde{T_{n}}$*, $\tilde{L_{n}}$*) shown in the Figure A and Figure B. For a given point, ($\tilde{T_{n}} ,\tilde{L_{n}}$), its coordinate can be rewritten as:

${\tilde{T_{n}}}^{'}= \tilde{T_{n}}$ -$\tilde{T_{n}}$*

${\tilde{L_{n}}}^{'}= \tilde{L_{n}}$- $\tilde{L_{n}}$*

At the same time, a ray oP (GEM) is defined by the operating point and the origin of the $T_{n}-o-L_{n}$ coordinate system (Figure B).

Second, as shown in Figure C, we calculate the coordinate of the ${\tilde{{(T}_{n}}}^{'},{\tilde{L_{n}}}^{'}$) in a new Cartesian coordinate system, which originates at the point P, with one axis along the oP direction and the other one, Pt, is perpendicular to oP.

Because the angle between oP and $T_{n}$ is θ, the coordinates of point ${\tilde{{(T}_{n}}}^{'},{\tilde{L_{n}}}^{'}$) in the new coordinate system, ($\delta_{p}$, $\delta_{T}$), can be calculated through rotation of the coordinate system using:

$\left[ \begin{matrix} \delta_{T} \\ \delta_{p} \end{matrix} \right]= R\left( \theta\right)\left[ \begin{matrix} {\tilde{T_{n}}}^{'} \\ {\tilde{L_{n}}}^{'} \end{matrix} \right]$ , where $R\left( \theta\right)= \left[ \begin{matrix} cos\theta& sin\theta\\ -sin\theta& cos\theta\end{matrix} \right]$ is a rotation matrix.

By taking the cos$\theta$ out from the rotation matrix, we will have:

$\left[ \begin{matrix} \delta_{T} \\ \delta_{p} \end{matrix} \right]= \left[ \begin{matrix} cos\theta& sin\theta\\ -sin\theta& cos\theta\end{matrix} \right]\left[ \begin{matrix} {\tilde{T_{n}}}^{'} \\ {\tilde{L_{n}}}^{'} \end{matrix} \right]$ = cos$\theta\left[ \begin{matrix} 1 & tan\theta\\ -tan\theta& 1 \end{matrix} \right]\left[ \begin{matrix} {\tilde{T_{n}}}^{'} \\ {\tilde{L_{n}}}^{'} \end{matrix} \right]$

 We further define$v=tan\theta= \frac{\tilde{L_{n}}*}{\tilde{T_{n}}*};and then cos\theta= \frac{1}{\sqrt{1+{tan}^{2}\theta}}$

By replacing $tan\theta$ with v, we can get the Equation 4

$\left[ \begin{matrix} \delta_{T} \\ \delta_{p} \end{matrix} \right]= \frac{1}{\sqrt{1+v^{2}}} \left[ \begin{matrix} 1 & v \\ -v & 1 \end{matrix} \right] \left[ \begin{matrix} {\tilde{T_{n}}}^{'} \\ {\tilde{L_{n}}}^{'} \end{matrix} \right]$
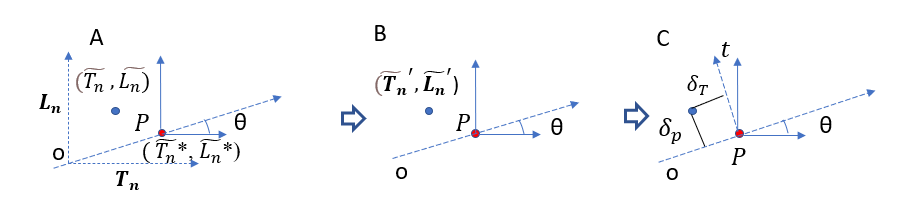


Figure 1. The demonstration of the process of coordination recenter (A -B) and rotation (C) for each step.
